# Mitigating Antibiotic Resistance Genes in Wastewater by Sequential Treatment with Novel Nanomaterials

**DOI:** 10.3390/polym13101593

**Published:** 2021-05-15

**Authors:** Lisa Paruch, Adam M. Paruch, Tanta-Verona Iordache, Andreea G. Olaru, Andrei Sarbu

**Affiliations:** 1 Division of Environment and Natural Resources, Norwegian Institute of Bioeconomy Research (NIBIO), Oluf Thesens 43, 1433 Aas, Norway; adam.paruch@nibio.no; 2 Advanced Polymer Materials and Polymer Recycling Group, National Institute for Research & Development in Chemistry and Petrochemistry ICECHIM, Splaiul Independentei 202, 060021 Bucharest, Romania; tanta-verona.iordache@icechim.ro (T.-V.I.); andr.sarbu@gmail.com (A.S.); 3 EDAS-EXIM S.R.L., Banat Street 23, 010933 Bucharest, Romania; andreea.olaru@edas.ro

**Keywords:** antibiotic resistance genes, emerging pathogens, nanomaterials, wastewater treatment

## Abstract

Wastewater (WW) has been widely recognized as the major sink of a variety of emerging pathogens (EPs), antibiotic-resistant bacteria (ARB) and antibiotic resistance genes (ARGs), which may disseminate and impact wider environments. Improving and maximizing WW treatment efficiency to remove these microbial hazards is fundamentally imperative. Despite a variety of physical, biological and chemical treatment technologies, the efficiency of ARG removal is still far from satisfactory. Within our recently accomplished M-ERA.NET project, novel functionalized nanomaterials, i.e., molecularly imprinted polymer (MIP) films and quaternary ammonium salt (QAS) modified kaolin microparticles, were developed and demonstrated to have significant EP removal effectiveness on both Gram-positive bacteria (GPB) and Gram-negative bacteria (GNB) from WW. As a continuation of this project, we took the further step of exploring their ARG mitigation potential. Strikingly, by applying MIP and QAS functionalized kaolin microparticles in tandem, the ARGs prevalent in wastewater treatment plants (WWTPs), e.g., *bla*CTXM, *erm*B and *qnr*S, can be drastically reduced by 2.7, 3.9 and 4.9 log (copies/100 mL), respectively, whereas *sul*1, *tet*O and *mec*A can be eliminated below their detection limits. In terms of class I integron-integrase I (*intI*1), a mobile genetic element (MGE) for horizontal gene transfer (HGT), 4.3 log (copies/100 mL) reduction was achieved. Overall, the novel nanomaterials exhibit outstanding performance on attenuating ARGs in WW, being superior to their control references. This finding provides additional merit to the application of developed nanomaterials for WW purification towards ARG elimination, in addition to the proven bactericidal effect.

## 1. Introduction

Wastewater treatment systems have turned out to be hotspots of antimicrobial resistance (the abilities of different microorganisms to resist antimicrobial agents) worldwide, particularly the municipal WWTPs receiving both industrial and domestic effluents [1,2,3,4,5,6,7,8]. The discharge of the undertreated WW can disseminate this resistance to wider natural environments, e.g., waters and soils; further, it can enter the food chain, endangering human and environmental health. Both ARB (bacteria resistant to at least one drug that used to inhibit or destroy these bacteria) and ARGs (genes resulting from genetic material mutation causing antibiotic resistance of a microorganism and/or genes acquired from an antibiotic-resistant microbe of the same or unrelated species) have been recognized as emerging contaminants in the environment [1,2,3]. In fact, genes associated with all classes of antibiotics and MGEs (i.e., plasmids, transposons, bacteriophages and integrons) are detected in WWTPs worldwide [1]. In an investigation of 12 urban WWTPs in seven European countries (Portugal, Spain, Ireland, Cyprus, Germany, Finland and Norway), it was found that ARGs conferring resistance to *β*-lactams (*bla*GES, *bla*OXA and *bla*VEB), macrolides (*ere*A and *erm*F), sulfonamides (*sul*1), tetracyclines (*tet*M and *tet*Q), aminoglycosides (*aad*A and *str*B), multidrug resistance (*qacEdelta*1 and *qac*H) and MGEs, e.g., integrases (*intI*1) and transposases (*tnp*A), were persistent in WWTP influent and effluent [2]. Similarly, a retrospective study on data concerning ARG prevalence in WWTPs across Europe, America, Asia and Africa from 2007–2019 was conducted by Wang et al. [3]; they concluded that ARGs resistant to *β*-lactam (*bla*CTXM, *bla*TEM, *bla*OXA-A, *bla*SHV and *mec*A), quinolone (*qnr*S, *qnr*C and *qnr*D), sulfonamide (*sul*1, *sul*2 and *dhfr*1), tetracycline (*tet*A, *tet*B, *tet*E, *tet*G, *tet*H, *tet*S, *tet*T and *tet*X), macrolide (*ere*A, *erm*B, *erm*C and *erm*43) and class 1 integron (*intI*1) were commonly detected in WWTPs. Moreover, *bla*CTXM, *bla*TEM, *sul*1, *sul*2, *tet*O, *tet*Q, *tet*W and *erm*B were identified as the dominant ARGs, being the most frequently reported.

Different treatment methods have been applied in an attempt to eliminate ARB (from both GNB and GPB) and ARGs from WW, such as ozonation and UV radiation [4], chlorination and UV disinfection [5], advanced oxidation processes [6] and membrane bioreactors [7], to name a few. However, ARG treatment efficiencies in real-scale WWTPs were observed at low degrees, namely 2–3 logs reduction, and in some cases, no reduction was registered [1]. Besides, divergent responses to the treatment were also noticed, implying that only limited types of ARGs are being inactivated [1]. Therefore, further optimization of operating conditions is required to obtain adequate ARG removal efficiency. However, increasing the usage doses of UV light, ozone and chlorine in order to maximize the efficiency raises concerns regarding the huge energy cost and excess hazardous chemical residues [8]. With the advancement of nanotechnology, the use of nanomaterials for water and WW treatment has been widely adopted in recent years [9]. Ma et al. [10] used nanosilver and silver ions to eliminate ARGs from WW. Titanium dioxide (TiO_2_) nanoparticles have shown antimicrobial activities through photocatalysis and, when combined with the use of chlorination and UV, the target ARGs in WW were inactivated [11]. Notably, carbon-based nanomaterials such as carbon nanotubes could effectively remove pathogens from WW due to their antimicrobial properties [12]; thus, they are promising for targeting ARGs owing to their remarkable pore structure, which facilitates effective adsorption and retention.

In this study, the newly developed nanomaterials as derived from a recently accomplished M-ERA.NET project were used for the trial on ARG mitigation in WW sampled from the influent of a WWTP. These nanomaterials include lipopolysaccharide (LPS) imprinted polymer films (LPS-MIP) and quaternary ammonium-functionalized-kaolin microparticles (QAS-K), which were designed for specifically targeting GNB and GPB, respectively. LPS constitutes the major component of the outer membrane of GNB and has been used for molecular imprinting of different nanoproducts for specific recognition and capture of whole bacteria [13,14]. The developed materials have been exploited in tandem to purify WW and demonstrated potent EP removal effects determined by culture-dependent (microbiological) and culture-independent (molecular) diagnostics [15]. Based on the achieved proven bactericidal activity exerted by the novel nanoproducts, we set out to answer a research question: To what extent do these nanomaterials mitigate ARGs? Following this query, we hypothesize that the novel nanoproducts can be functionally active and applicable for the removal of ARGs from WW, since a considerable number of antibiotic resistance carriers, i.e., ARB (particularly pathogenic ARB), can be removed/inactivated through the treatment. To address this hypothesis, we examined and quantified seven ARGs, representing the prevalent ARGs frequently detected in WWTPs, and HGT-related *intI*1 in WW before and after treatment with the novel nanomaterials using genetic marker-based quantitative real-time PCR (qPCR). Individual ARG reduction was estimated from the analyzed quantitative data.

## 2. Materials and Methods

### 2.1. Materials

Two types of functionalized nanomaterials were generated in film and microparticle forms. For the film format, LPS, a toxic component of the outer membrane of GNB, was used as a template to produce LPS-MIP using the molecular imprinting method, which was designed for specific GNB affinity to the imprinted cavity. Additionally, for the purpose of parallel comparison, non-imprinted polymer (NIP) control films were synthesized without LPS being added. For the other microparticle format, a commercial kaolin was made to undergo stepwise functional modifications to attain grafting with antimicrobial QAS. The resulting nanocomposite was denoted as QAS-K. The specific technical details about the preparation and characterization of these materials can be retrieved from our previous studies [15]. Briefly, four treatment formats were investigated, namely MIP-T (LPS-MIP in tandem with QAS-K), NIP-T (NIP in tandem with QAS-K), MIP-K (MIP with kaolin) and NIP-K (NIP with kaolin).

For molecular assays, One Shot™ OmniMAX™ 2 T1R Chemically Competent *E. coli* (Thermo Fisher Scientific, Waltham, MA, USA) was used for the cloning of the seven target ARG markers (i.e., *bla*CTXM, *erm*B, *qnr*S, *sul*1, *tet*O, *mec*A and *van*A) and *intI*1, and the sequences of the yielded clones were validated and confirmed by DNA sequencing at Eurofins Genomics Germany GmbH (Ebersberg, Germany). The primers and TaqMan probe for each ARG qPCR assay were prepared at Thermo Fisher Scientific (Waltham, MA, USA) and the sequence information is shown in Table 1. The applied microbial genomic DNA (gDNA) was derived from the previous treatment experiment using WW sampled in February 2020 and it was stored at −80 °C since then. The DNA concentration and integrity were re-validated on a mySPEC Spectrophotometer (VWR, Radnor, PA, USA). The details regarding the sampling campaign, ultrafiltration and gDNA recovery were described explicitly in [15]. SsoAdvanced™ Universal Probes Supermix (Bio-Rad Laboratories, Irvine, CA, USA) was used for the standard qPCR setup.

### 2.2. Establishment and Validation of ARG Markers

The target ARGs for this study were selected based on a thorough literature review on recent (from the last five years) publications regarding the ARGs frequently detected in WWTPs in most countries in Europe [2]. The genomic region for the amplicon of each individual marker was screened and verified in silico to attain maximal qPCR sensitivity and specificity. The designed marker genes were chemically synthesized (GenScript, Leiden, The Netherlands) and confirmed by sequencing. The target gene carrying plasmid was cloned into *E. coli* K12 OmniMAX™ 2 T1R following the protocol provided (Thermo Fisher Scientific, Waltham, MA, USA). The final construct was verified by sequencing with 100% sequence identity and used for the establishment of each qPCR assay. The individual qPCR program for each ARG marker was tested, optimized and validated using the serial dilutions of each gene construct. The assays with 95–100% amplification efficiency and above 0.99 linearity of the resulting standard curve were achieved with detection limits at 1–10 copies per reaction.

### 2.3. qPCR Analysis of ARG Abundance in Wastewater

The detection and quantification of the target ARG was performed in duplicate on a Bio-Rad CFX Connect Real-Time PCR Detection System (Irvine, CA, USA). In 20 µL qPCR reaction, 10 µL of SsoAdvanced™ Universal Probes Supermix was mixed with 500 nM of each primer, 250 nM 5′-FAM probe and sterile nuclease-free H_2_O. The amplification conditions were as follows: 95 °C for 3 min, followed by 40 cycles of 95 °C for 15 s and 60 °C for 30 s. A standard calibrating curve was established using 10-fold serial dilutions of plasmids carrying the marker gene (from 10^6^ to 10^0^ copies/µL). The quantification using CFX Manager Version 3.1 (Bio-Rad, Irvine, CA, USA) was performed on the raw data derived from the qualified assay (i.e., amplification efficiency ranging from 90–100% and regression rate above 0.99).

## 3. Results and Discussion

Among all the tested ARGs, *van*A was not detected in the raw WW samples (and as expected, it was also not detected in the treated samples using different nanomaterials) and *mec*A was found at a low level (3.5 log copies/100 mL). *van*A and *me*cA confer resistance to vancomycin and methicillin, respectively, which are considered last-resort antibiotics [21]. Therefore, the absence of *van*A and the low level of *mec*A in the examined WWTP, which were similar to another finding [22], are particularly attributed to their limited discharge with WW; hence, they have low loads in the influents of treatment systems. The WWTP dominant ARGs, e.g., *erm*B, *qnr*S, *sul*1 and *tet*O, were found at high levels in the raw WW, at 6.4, 10.2, 6.9, and 7.6 log copies/100 mL, respectively (Table 2). Class 1 integron *intI*1 was also detected with the high abundance of 7.4 log copies/100 mL.

**Figure 1:**
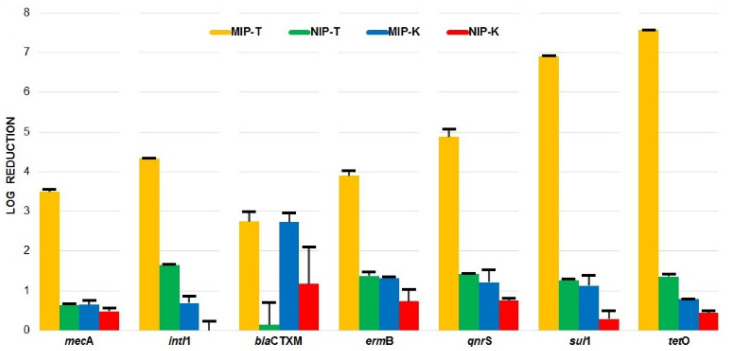
ARG log reductions after treatment of WW with the novel nanomaterials, namely MIP-T (LPS-MIP in tandem with QAS-K), NIP-T (NIP in tandem with QAS-K), MIP-K (MIP with kaolin) and NIP-K (NIP with kaolin).

The treatment application using LPS-MIP films and QAS-K in tandem (MIP-T) exhibited superior ARG removal efficacy in comparison to their references, i.e., non-LPS imprinted films and non-QAS-K microparticles (NIP-T, MIP-K and NIP-K). As shown in Figure 1, the log reductions achieved by the developed nanomaterial combination (MIP-T) were at the high levels of 4.3 (*intI*1), 2.7 (*bla*CTXM), 3.9 (*erm*B) and 4.9 (*qnr*S). It is worth noting that no detectable *mec*A, *sul*1 and *tet*O were found in MIP-T-treated WW, indicating an adequate removal accomplishment by the synergy of MIP-T. Such a performance is quite encouraging for applying the novel nanomaterials to WW treatment, since *sul*1 and *tet*O are among the most frequently found and dominant ARGs in different WWTPs due to the common and extensive use of their associated antibiotics, i.e., Sulfonamide and Tetracycline, in medical, industrial and agricultural areas [23]. In particular, *sul*1 was found to be difficult to remove by conventional treatments [22,24]. In the case of class 1 integron *intI*1, as a member of MGE, it is responsible for the HGT of ARGs across various bacterial species and other organisms, since many ARGs are transferred as integrons; e.g., 85% of *sul*1 and 76% of *sul*2 were detected to be associated with integrons in riverine systems [25]. Moreover, considering its close link to antimicrobial resistance, disinfectants and heavy metals associated directly with human impacts, it was recommended as a proxy for anthropogenic pollution [26] and served as a monitoring indicator for the elimination of ARGs in WWTPs [27]. In this context, the achieved 4.3 log reduction of *intI*1 in WW by the novel nanomaterials evidenced a robust treatment efficiency towards ARG inactivation.

The identified ARG mitigation efficacy elicited by the applied new nanoproducts can be associated with the substantial pathogen inactivation in the tested WW, as revealed in our previous study [15]. Evidently, there are preferential co-occurrence patterns/hosting relationships between ARGs and the bacterial pathogens [28]. Our earlier study, carried out on the same nano-based treatment formats, indicated that waterborne pathogenic bacteria (including both GNB and GPB), such as *Salmonella typhimurium* (*S. typhimurium*), *Enterococcus faecalis* (*E. faecalis*), *Clostridium perfringens* (*C. perfringens*), Shiga-toxin producing *E. coli* and *Campylobacter jejuni* (*C. jejuni*), were efficaciously eliminated from WW [15]. These bacterial pathogens are found to be closely related to ARGs such as *bla*CTXM (*S. typhimurium, E. faecalis* and *E. coli*), *erm*B (*E. faecalis* and *C. perfringens*), *qnr*S (*E. coli, S. typhimurium* and *Shigella spp*.), *sul*1 (*E. coli, Salmonella spp.* and *Shigella spp*.) and *tet*O (*C. jejuni*). Thus, one of the possible mechanisms for the displayed ARG inactivation can be ascribed to the large removal of the ARG-associated pathogens. Numerous nano-based WW treatment methods rely mainly on efficient ARB elimination to achieve ARG reduction [1]. Beyond that, distinct physicochemical properties possessed by nanostructured materials, such as surface effects and high porosity for the adsorption of small molecules, e.g., DNA [29], are among the other possible modes of action. It was also reported that, in the presence of ammonium groups, the nucleic acids (e.g., DNA) can bind strongly to mesoporous silica [30]. The developed hybrid microparticles were prepared from the monodisperse clay beads functionalized with polycationic brushes and QAS bearings, which can facilitate the binding of the anionic DNA of ARGs and thus augment their retention and removal from WW.

## 4. Conclusions

In summary, the examination of the tandem application of LPS-MIP films and QAS-K microparticles for removing ARGs from WW managed to address the defined research question and proved the hypothesis was correct/true. The results from the qPCR analysis on the selected ARGs manifested a superior performance of such an application, with a remarkable reduction of the ARG load in treated WW. These outcomes, combined with the previously demonstrated bactericidal effect on EP removal, highlight that the newly developed nanomaterials have profound potential to be integrated as an innovative solution in tackling wastewater-derived antibiotic resistance. Further investigations on larger scale treatment systems and their functional dynamics (e.g., time kinetic, under variable reaction conditions, dose-dependent effect, etc.) will be devised and pursued.

## Figures and Tables

**Table 1:** ARG markers used in the assay with sequence information of primers (F, forward primer; and R, reverse primer) and probes (P, TaqMan probe).

ARG Markers	Resist to Antibiotics/Class	Sequences of Primes and Probes (5′–3′)	Ref.
*mec*A	Methicillin, Penicillin/β-lactam	F: CATTGATCGCAACGTTCAATTTAAT	[16]
		R: TGGTCTTTCTGCATTCCTGGA	
		P: FAM-CTATGATCCCAATCTAACTTCCACATACC-MGBNFQ	
*erm*B	Erythromycin/Macrolides	F: GGATTCTACAAGCGTACCTTGGA	[16]
		R: GCTGGCAGCTTAAGCAATTGCT	
		P: FAM-CACTAGGGTTGCTCTTGCACACTCAAGTC-MGBNFQ	
*qnr*S	Fluoroquinolones	F: CGACGTGCTAACTTGCGTGA	[17]
		R: GGCATTGTTGGAAACTTGCA	
		P: FAM-AGTTCATTGAACAGGGTGA-MGBNFQ	
*sul*1	Sulfonamide	F: CCGTTGGCCTTCCTGTAAAG	[18]
		R: TTGCCGATCGCGTGAAGT	
		P: FAM-CAGCGAGCCTTGCGGCGG-MGBNFQ	
*tet*O	Tetracycline	F: AAGAAAACAGGAGATTCCAAAACG	[16]
		R: CGAGTCCCCAGATTGTTTTTAGC	
		P: FAM-ACGTTATTTCCCGTTTATCACGGAAGCG-MGBNFQ	
*van*A	Vancomycin	F: CTGTGAGGTCGGTTGTGCG	[19]
		R: TTTGGTCCACCTCGCCA	
		P: FAM-CAACTAACGCGGCACTGTTTCCCAAT-MGBNFQ	
*bla*CTXM	Cefotaxime, Ceftazidime/β-lactam	F: ACCAACGATATCGCGGTGAT	[17]
		R: ACATCGCGACGGCTTTCT	
		P: FAM-TCGTGCGCCGCTG-MGBNFQ	
*intI*1	Multidrug resistance	F: GCCTTGATGTTACCCGAGAG	[20]
		R: GATCGGTCGAATGCGTGT	
		P: FAM-ATTCCTGGCCGTGGTTCTGGGTTTT-MGBNFQ	

**Table 2:** qPCR quantified ARG abundance in copy numbers with standard error of means in 100 mL of WW, untreated and treated with the novel nanomaterials, namely MIP-T (LPS-MIP in tandem with QAS-K), NIP-T (NIP in tandem with QAS-K), MIP-K (MIP with kaolin) and NIP-K (NIP with kaolin).

Samples	*intI*1	*bla*CTXM	*erm*B	*qnr*S	*mec*A	*sul*1	*tet*O
WW	2.35 × 10^7^ ± 9.49	7.40 × 10^4^ ± 3.76 × 10^−1^	2.71 × 10^6^ ± 1.14 × 10^1^	1.67 × 10^10^ ± 2.74 × 10^4^	3.47 × 10^3^ ± 6.67 × 10^−2^	8.14 × 10^6^ ± 3.08 × 10^1^	3.69 × 10^7^ ± 1.31 × 10^2^
MIP-T	1.09 × 10^3^ ± 3.03 × 10^−3^	1.03 × 10^2^ ± 4.54 × 10^−3^	3.54 × 10^2^ ± 4.61 × 10^−2^	1.76 × 10^5^ ± 7.07	0.00 ± 0.00	0.00 ± 0.00	0.00 ± 0.00
NIP-T	5.13 × 10^5^ ± 6.51	2.87 × 10^4^ ± 7.74	1.02 × 10^5^ ± 1.51	6.16 × 10^8^ ± 4.90 × 10^3^	7.40 × 10^2^ ± 1.03 × 10^−2^	4.21 × 10^5^ ± 8.21	1.51 × 10^6^ ± 6.94 × 10^1^
MIP-K	3.91 × 10^6^ ± 8.47 × 10^1^	1.12 × 10^2^ ± 3.29 × 10^−2^	1.26 × 10^5^ ± 3.72	7.19 × 10^8^ ± 7.21 × 10^3^	6.96 × 10^2^ ± 7.67 × 10^−2^	4.57 × 10^5^ ± 8.03 × 10^1^	5.92 × 10^6^ ± 4.22 × 10^1^
NIP-K	2.07 × 10^7^ ± 4.03 × 10^2^	1.98 × 10^3^ ± 9.42 × 10^−1^	3.56 × 10^5^ ± 1.30 × 10^1^	2.71 × 10^9^ ± 7.61 × 10^−1^	9.90 × 10^2^ ± 3.05 × 10^−2^	3.39 × 10^6^ ± 1.17 × 10^2^	1.27 × 10^7^ ± 5.84 × 10^2^

## Data Availability

Data is contained within the article.
